# Supplementary material for: High prevalence of injection drug use and blood-borne viral infections among patients in an urban emergency department
Source: PLoS One. 2020 Jun 4;15(6):e0233927. doi: 10.1371/journal.pone.0233927 (PMC7272082; doi:10.1371/journal.pone.0233927)
Supplement: S1 Appendix — (PDF) [file pone.0233927.s001.pdf]

STUDY ID:
IRB18-XXXXXX
Version Date: 07/15/2018

SURVEYOR INITIALS: ______________________________

DATE:_________________

Patient Sticker Here

Name
DOB
Medical Record number

Survey Start: All answers are patient self-report

1. Have you ever been diagnosed with hepatitis C? Do you have hepatitis C?
   - ☐ Yes
   - ☐ No
   - ☐ Don't know
   - ☐ Prefer not to answer

   1b. If yes, have you received treatment for your hepatitis C?
   - ☐ Yes
   - ☐ No
   - ☐ Don't know
   - ☐ Prefer not to answer

   1c. If yes to treatment.
   - ☐ Treatment in progress
   - ☐ Cured
   - ☐ Not cured
   - ☐ Don’t know
   - ☐ Prefer not to answer

2. Have you ever been diagnosed with HIV? Do you have HIV?
   - ☐ Yes
   - ☐ No
   - ☐ Don't know

   2b. If yes, are you in currently receiving care for your HIV?
   - ☐ Yes
   - ☐ No
   - ☐ Don't know
   - ☐ Prefer not to answer

3. Have you ever used any drug by injection (non-medical use only)? *An example of a medical use for injecting a drug would be injecting insulin to manage your diabetes. We’re talking about injecting drugs like heroin, cocaine, meth.*
   - ☐ Yes
   - ☐ No
   - ☐ Don't know
   - ☐ Prefer not to answer

   3b. If yes, When was the last time you injected?
   - ☐ In the past 90 days
   - ☐ In the past year
   - ☐ Over a year ago
   - ☐ Don't know
   - ☐ Prefer not to answer
